# An age-period-cohort analysis of hysterectomy incidence trends in Germany from 2005 to 2019

**DOI:** 10.1038/s41598-024-66019-8

**Published:** 2024-07-02

**Authors:** Gifty Baffour Awuah, Gunther Schauberger, Stefanie J. Klug, Luana Fiengo Tanaka

**Affiliations:** Chair of Epidemiology, TUM School of Medicine and Health, Technical University of Munich, Georg-Brauchle-Ring 56, 80992 Munich, Germany

**Keywords:** Epidemiology, Epidemiology

## Abstract

Recent studies show declining trends in hysterectomy rates in several countries. The objective of this study was to analyse hysterectomy time trends in Germany over a fifteen-year period using an age-period-cohort approach. Using an ecological study design, inpatient data from Diagnoses Related Group on hysterectomies by subtype performed in Germany from 2005 to 2019 were retrieved from the German Statistical Office. Descriptive time trends and age-period-cohort analyses were then performed. A total of 1,974,836 hysterectomies were performed over the study period. The absolute number of hysterectomies reduced progressively from 155,680 (365 procedures/100,000 women) in 2005 to 101,046 (257 procedures/100,000 women) in 2019. Total and radical hysterectomy decreased by 49.7% and 44.2%, respectively, whilst subtotal hysterectomy increased five-fold. The age-period-cohort analysis revealed highest hysterectomy rates in women aged 45–49 for total and subtotal hysterectomy with 608.63 procedures/100,000 women (95% CI 565.70, 654.82) and 151.30 procedures/100,000 women (95% CI 138.38, 165.44) respectively. Radical hysterectomy peaked later at 65–69 years with a rate of 40.63 procedures/100,000 women (95% CI 38.84, 42.52). The risk of undergoing total or radical hysterectomy decreased over the study period but increased for subtotal hysterectomy. Although, overall hysterectomy rates have declined, subtotal hysterectomy rates have increased; reflecting changes in clinical practice largely influenced by the availability of uterus-sparing options, evolving guidelines and introduction of newer surgical approaches.

## Introduction

Globally, hysterectomy is the most frequent gynaecological surgery^1^. In Germany, the prevalence of hysterectomy between 2008 and 2011 in 18–79-year-olds was 18%^2^. In 2021 alone, an estimated 90,000 inpatient hysterectomies were performed^3^, making it the third most common inpatient gynaecological surgery in the country. Nearly 90% of indications for hysterectomy are for benign conditions^4,5^. The most frequent being uterine fibroids, representing 60% of all in-patient hysterectomies performed in 2012^4^, followed by abnormal uterine bleeding and pelvic prolapse. Of the malignant indications, endometrial cancer ranked first.

Over the past decades, there has been a proliferation of non-surgical treatments for benign gynaecological diseases^6–10^. Coincidentally, the rates of hysterectomy are reportedly decreasing in several countries. In analysing trends from 2006 to 2012 in Germany, a similar declining pattern was found, indicating a shift towards more conservative procedures^11^. However, when examining subtypes, the subtotal hysterectomy rates had increased.

The age-period-cohort analysis is a tool for analysing trends of population-level data aiming at disentangling the effects of age, period and cohort on the changes in the trends^12^. Age effects are those due to the intrinsic biological process of aging; period effects reflect changes associated with time, affecting all age groups simultaneously. Cohort effects are limited to particular cohorts because of specific exposures at a point in time^13^. Although commonly used in cancer studies, there has been recent use of this approach for diagnostic procedures as well^14,15^.

The objective of this study was to examine the age, period and cohort effects on trends of overall hysterectomies and subtypes in Germany based on nationwide Diagnoses Related Group data from 2005 to 2019.

## Materials and methods

Using an ecological study design, population-level data was used to examine the trends of hysterectomy incidence in Germany. The introduction of the German Diagnoses Related Group remuneration system for inpatient services in 2004, which demands reporting of all inpatient services for reimbursement, enables the analysis of national trends in inpatient hysterectomy from 2005 onwards. Nationwide Diagnoses Related Group hysterectomy data was obtained using the following German Operation and Procedure Classification (OPS) codes: Subtotal hysterectomy: 5–682, total hysterectomy: 5–683, obstetric hysterectomy: 5–757 and radical hysterectomy: 5–685. This data is publicly available from the German Statistical Office^16^. The yearly population at risk, i.e. Germany’s female population from 2005 to 2019 by 5-year age group, was also retrieved from the German Statistical Office^17^.

Crude incidence rates were calculated with the absolute number of hysterectomies as the numerator and the denominator as the annual female population obtained from the Federal Statistical Office (per 100,000). Trends of all hysterectomies and the subtypes except for obstetric hysterectomy were visualised with trend graphs. Except for calculating the rate of all hysterectomies, obstetric hysterectomy was not included in the analyses because it is less frequently performed and limited to the child-bearing age group. Analyses by age group as well as age-period-cohort were restricted to the ages 20 years and above because of the rarity of hysterectomy in younger women.

For the age, period, cohort analysis, age and period were grouped in five-year intervals. Fourteen age groups were obtained, the first being the 20–24-year group and the last group consisting of people aged 85 years and above, but for this study, it was taken as a 5-year age group, 85–89. Three period groups were obtained: 2005–2009, 2010–2014 and 2015–2019. The midpoint of age and period were used to represent each group. The packages used created sixteen cohorts from 1920–1924 to 1995–1999 by subtracting the age from the period.

Descriptive plots were first created with the help of the rateplot function of the Epi R package for the three hysterectomy subtypes. Four classical plots for each data set were then produced: Age-specific rates by period, age-specific rates by date of birth, period-specific rates by age and cohort-specific rates by age.

The age period cohort effects were estimated using the National Cancer Institute APC web tool (available at /https://analysistools.cancer.gov/apc/). For our results, we focused mainly on the estimated functions: age-specific rates, period- and cohort-specific rate ratios (RR), local drifts (annual percentage changes for each age group) and the net drifts (indicating the overall annual percentage change). The decision on whether to report the age-specific longitudinal or age-specific cross-sectional rates was made after careful analysis of the descriptive plots utilising the theory proposed by Clayton^18^. This theory presupposes the presence of a strong period effect when “parallelism”—the consistent variation in the single curves—is present in the age-specific rates by period plot. The absence of parallelism suggests predominant cohort effects. The reference period was the median year for 2010 to 2014; 2012 and the reference cohort 1956–1960. The significance of estimable functions was determined using Wald Chi-square tests. The statistical significance level was set at 0.05.

### Ethics approval

This study used publicly published secondary data with no individual data; therefore no ethical approval was needed for this study.

## Results

**Figure 1:**
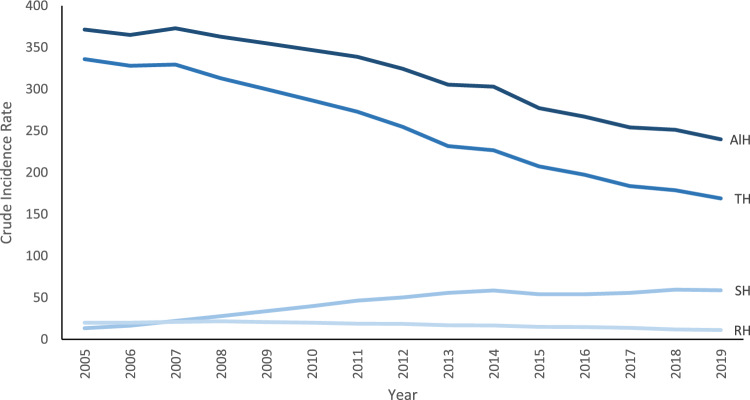
Crude incidence rate of hysterectomies performed by type. Germany, 2005–2019. *AlH* All hysterectomies, *TH* Total hysterectomy, *SH* Subtotal hysterectomy, *RH* Radical hysterectomy.

A total of 1,974,836 inpatient hysterectomies were performed in Germany from 2005 to 2019. Overall, the hysterectomy rate reduced from 371.53 procedures per 100,000 women per year in 2005 to 239.85 procedures per 100,000 women per year in 2019. Total hysterectomy remains the most common type of hysterectomy performed in Germany, although the total numbers and proportions have decreased over time. Total hysterectomy rates decreased from 335.97 per 100,000 women in 2005 to 168.99 per 100,000 women in 2019. The radical hysterectomy rates and numbers also decreased over the period, but the proportions remained unchanged. In contrast, the numbers, proportions and rates of subtotal hysterectomy increased over the period; from 6.2% of all hysterectomies and a crude rate of 22.67 per 100,000 women in the 2005–2009 period to almost 21.9% and a rate of 56.50 per 100,000 women in the 2015–2019 period (Table 1 and Fig. 1).

**Table 1:** Number and crude rates of hysterectomies by subtypes. Germany, 2005–2019*.*

Period	Total		Subtotal		Radical		Obstetric		All	
	N (%)	CR*	N (%)	CR*	N (%)	CR*	N (%)	CR*	N (%)	CR*
2005–2009	673,342 (87.9)	321.29	47,550 (6.2)	22.67	43,433 (5.7)	20.72	907 (0.1)	0.43	765,232	365.49
2010–2014	525,707 (78.7)	254.64	103,659 (15.5)	50.21	37,529 (5.6)	18.18	1467 (0.2)	0.71	668,362	323.74
2015–2019	392,486 (72.6)	187.24	118,435 (21.9)	56.50	27,911 (5.2)	13.32	1682 (0.3)	0.80	540,514	257.86
Total	1,591,535 (80.6)	254.39	269,644 (13.7)	43.10	108,873 (5.5)	17.40	4,056 (0.2)	0.65	1,974,108	315.65

*N* Number, *%* Percentage of all hysterectomies, *CR* Crude rate.

*Rate per 100,000 women.

**Figure 2:**
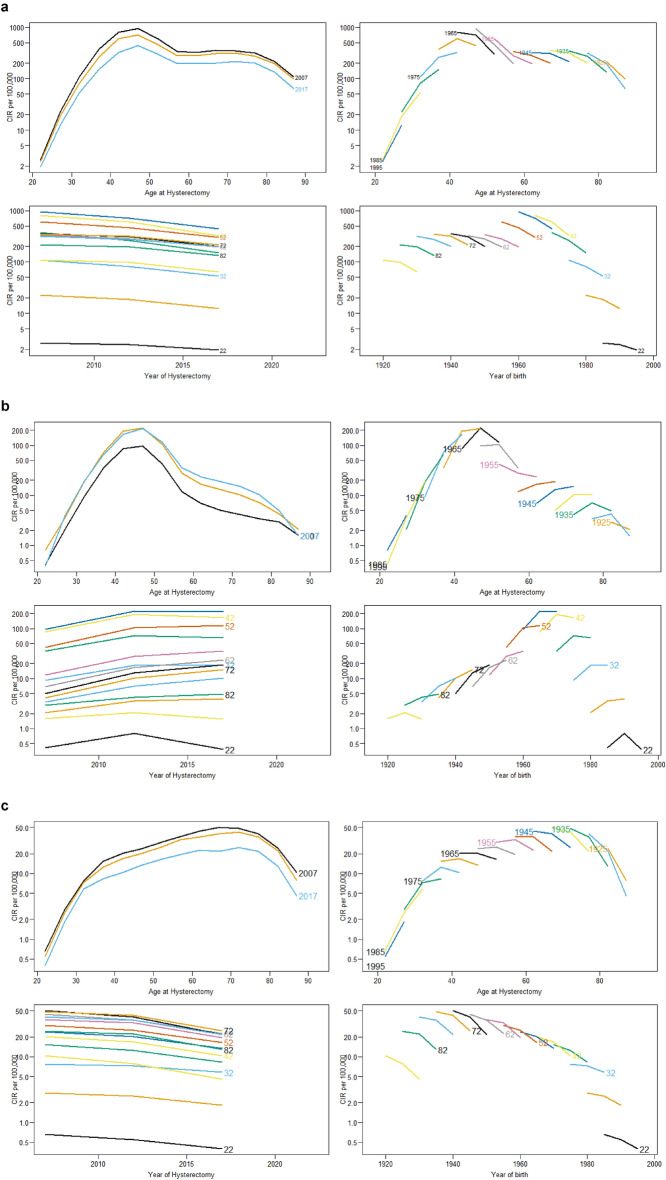
Crude rates of hysterectomies subtypes by age and period, age and cohort, period and age and cohort and age. Germany, 2005–2019. *CIR* Crude incidence rate.

In the descriptive graphs for total and radical hysterectomy, the age-specific rates by period plot show a consistent trend across the three periods, namely, 2005–2009, 2010–2014 and 2015–2019 (Fig. 2). There is a larger decline between the 2010–2014 and 2015–2019 periods than between the 2005–2009 and 2010–2014 periods. In contrast, the subtotal hysterectomy graph reveals a reversal in this trend, with increasing rates across the periods. The increase in rates is also higher in the 2005–2009 and 2010–2014 periods compared to 2010–2014 and 2015–2019 periods. The age-specific rates by period graph and age-specific rates by birthdate demonstrate a consistent morphology between the plots and across the subtypes. Since all the age-specific rates by period graphs are similar in all the three subtypes, the age-specific cross-sectional rates are reported, where age effects are presented as the age-specific incidence rates for the reference period.

**Figure 3:**
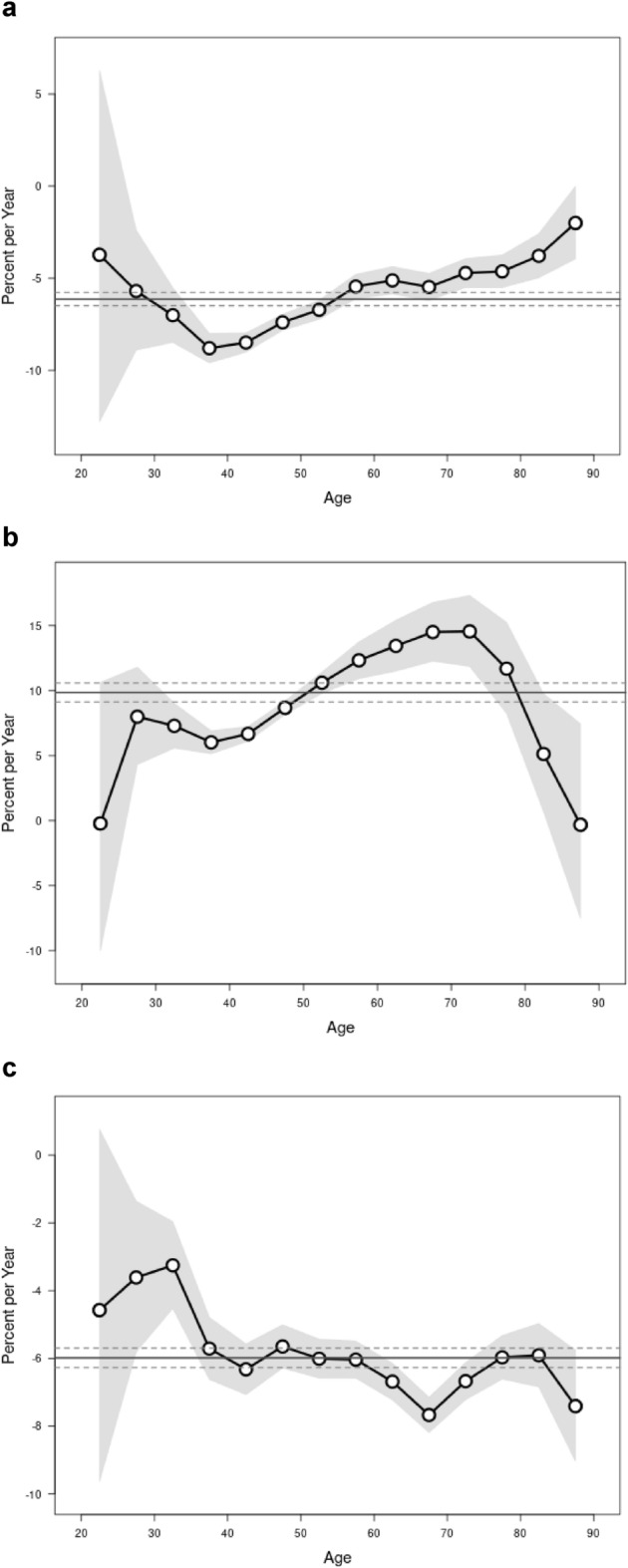
Net drift (annual percentage change in expected overall age adjusted rates) and local drift (expected age-specific rates over time) by hysterectomy type in Germany: 2005–2019. (**a**) total hysterectomy, (**b**) subtotal hysterectomy, (**c**) radical hysterectomy. The solid and dashed horizontal lines represents the net drift and the accompanying confidence intervals, the points and the shaded area represents the local drifts and the confidence intervals.

Figure 3 shows the (annual percentage change in expected overall age-adjusted rates) and the local drift (expected age-specific rates over time) by hysterectomy subtype. The overall net drift per year was − 6.13 (95% CI − 6.49, − 5.77) for subtotal hysterectomy, for subtotal hysterectomy 9.86 (95% CI 9.13, 10.58) and − 5.99 (95% CI − 6.27, − 5.70) for radical hysterectomy. Except for the 20–24-year age group, the local drifts are significant for all other age groups for all the hysterectomy subtypes. The local drifts decreased significantly for all age groups for total hysterectomy, with those age 60 years and above having the greatest decrease. Local drifts decreased significantly for radical hysterectomy, also, with the greatest decrease being in the 65–70-year age group. The local drifts increased in all age groups for subtotal hysterectomy, with the greatest drift being in the 55–80-year age group.

**Figure 4:**
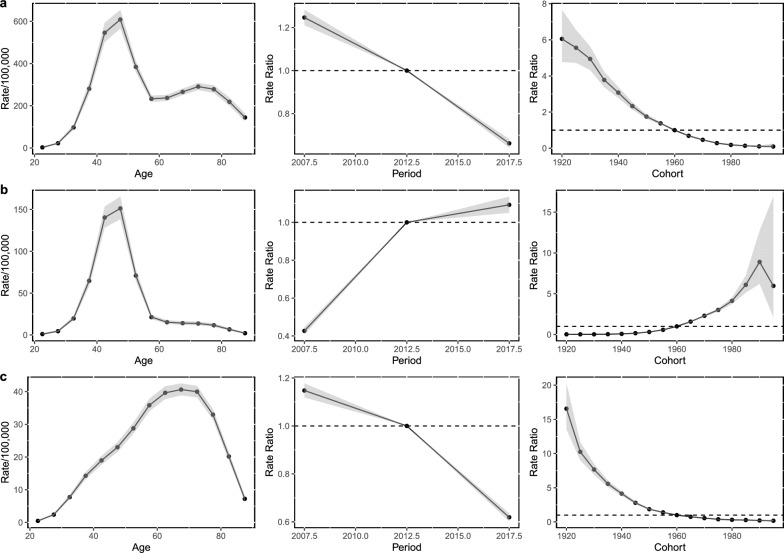
Age, period and cohort effects: Hysterectomy subtypes. Germany, 2005–2019. (**a**) total hysterectomy, (**b**) subtotal hysterectomy, (**c**) radical hysterectomy. Age effects are the expected age-specific rates in reference period adjusted for cohort effects, Ratio of age-specific rates in each period relative to reference period (2010–2014), Ratio of age-specific rates in each cohort relative to reference cohort (1956–1960).

Total hysterectomy rates increase steeply with increasing age until it peaks at 45–49 years with 608.63/100,000 women (95% CI 565.70, 654.82), then decrease until around age 60 years, and then increase slightly again in older years (Fig. 4a). The period effects show an increasing total hysterectomy risk over the period. The cohorts born after 1960 had a lower risk of having a total hysterectomy performed compared to those born before this period. Like total hysterectomy incidence rates, subtotal hysterectomy is rarely performed in younger women. Rates then increase steeply, peaking at age 45–49 with rates of 151.30/100,000 women (95% CI 138.38, 165.44), before decreasing rapidly again in the older age groups (Fig. 4b). The risk of undergoing a subtotal hysterectomy increased over the study period. The cohort born after 1960 had a higher risk of undergoing a subtotal hysterectomy compared to the cohorts born before 1960. Radical hysterectomy rates are almost zero at 20 years, increasing sharply until 50 years, and then the slope of increase becomes gradual, peaking at 65–69 years with 40.63/100,000 (95% CI 38.84, 42.52) and decreases after 75 years (Fig. 4c). The risk of radical hysterectomy has decreased with increasing birth cohort, with cohorts born after 1960 having a decreased risk of having radical hysterectomy performed. Supplementary Table 2 shows all model estimates and confidence intervals. All estimated functions (cohort and period rate ratios and net and local drifts) were statistically significant (p < 0.01) in all hysterectomy subtypes (Supplementary Table 1).

## Discussion

This study aimed to analyse the trends of all hysterectomy cases and subtypes performed in Germany and to estimate the age, period, and cohort effects using an age-period-cohort analysis. The analysis showed decreasing hysterectomy rates overall and for all subtypes except for subtotal hysterectomy, with a peak age of 45–49 years for total and subtotal hysterectomy and a later peak at 65–69 years for radical hysterectomy. All hysterectomy subtypes showed predominant period effects with decreasing risk over the study period, whilst subtotal hysterectomy showed a reverse pattern.

The decreasing hysterectomy incidence rates observed in this study are similar to trends in other European countries such as Austria^19^, Portugal^20^ and Poland^21^. In recent years, there has been a plethora of options available to treat benign conditions, such as the levonorgestrel-releasing intrauterine system for abnormal uterine bleeding, myomectomy, uterine artery embolisation and Magnetic Resonance-guided focused ultrasound surgery for fibroids, which have made invasive procedures such as hysterectomy a second choice for benign indications^9,10^. Indeed, the German Society of Obstetricians and Gynaecologists S3 guidelines released in 2015 recommend these conservative therapies as the first line for managing most benign indications, especially when fertility preservation is desired^8^. In managing pelvic prolapse, for example, routine hysterectomy should no longer be performed except when indicated.

With the availability of these uterus-sparing treatment options, several criticisms have emerged since the early 1990s over the possible overuse of hysterectomy to treat benign diseases^22^. A probable response to this is an intentional attempt to reduce the overuse of hysterectomy for benign conditions across all age groups, an effect that is seen as the reduced risks of having a total hysterectomy (the most performed type of hysterectomy) over the study period. In a Danish analysis of hysterectomy trends, the observed decreasing hysterectomy incidence trends between 2000 and 2015 were due to the decrease in hysterectomies performed for benign diseases^23^.

Hysterectomies are performed using either the vaginal approach, open abdominal approach or the more recent laparoscopic approach, which is now more commonly performed^24–26^. In Germany, a study at a tertiary facility looking at surgical approaches between 2007 and 2016 revealed that the use of abdominal hysterectomy decreased from 61.4 to 13.4%, laparoscopic hysterectomy increased from 4.1 to 69.7% and vaginal hysterectomy decreased to 14.6%, despite an initial increase from 21 to 45.5% between 2007 and 2013^24^. Under the laparoscopic approach, there are three subtypes, the total laparoscopic hysterectomy, laparoscopic subtotal hysterectomy and laparoscopic-assisted vaginal hysterectomy, which is a combination of the vaginal and laparoscopic approaches^25,26^.

The laparoscopic subtotal hysterectomy, compared to the total, was said to be relatively easier to perform, requiring shorter operating time because the difficult dissection around the cervix and bladder is not required with this procedure^25,27^. The procedure was, therefore, perceived to be associated with less bleeding, lower urinary tract complications and better pelvic floor integrity^27,28^. Initial case series and retrospective analyses also reported better sexual function with subtotal hysterectomy^29,30^. These reports and suggestions may have influenced the choice of surgical approach for hysterectomy, resulting in the increase in subtotal hysterectomy rates found in our analysis. However, in recent larger randomized trials and meta-analyses, the reported better sexual well-being, pelvic floor integrity, and urinary tract complications with subtotal hysterectomies could not be confirmed^31,32^. Although subtotal hysterectomy is still associated with less operating time and blood loss, this has not been found to be clinically significant^31,32^.

In 2014, the Food and Drug Administration warned against the risk of inadvertent spread of occult malignancy with the use of morcellators with the laparoscopic subtotal hysterectomy procedure^33^, which subsequently led to a 50% reduction in the procedure in the US^34^. Decreased conduct of the procedure has been reported in countries like Finland^35^ and Brazil^36^. In Germany, guidelines by the German Society of Obstetricians and Gynaecologists did not limit the use of this approach but recommended an investigative workup to exclude malignancy before the procedure^8^. However, one can still notice the effect of this warning by the relatively smaller increase in rates compared to the 2010–2014 period.

The high incidence of cervical cancer that led to the increase in the number of total hysterectomies in the mid-twentieth century^37^ has declined where population-based screening has been introduced. Additionally, the fear of the risk of cancer of the cervical stump that perpetuated this preference has also been reported not to be higher than the risk in the general population in areas where good screening measures are available and utilised post hysterectomy^38,39^. However, in a Danish study, having a subtotal hysterectomy after the age of 50 years was found to be associated with up to a five-fold increase in cervical cancer risk^39^. This could likely explain the rarity of the subtotal procedure in older women. The statutory cervical cancer screening program, which offers regular testing to women aged 20 years and older, was instituted in 1971 in West Germany and expanded to the East in 1994^40^. This has resulted in a higher rate of diagnosis of precancerous lesions and early-stage cervical cancer^41^. Early diagnosis enables the use of less invasive surgeries such as conisation and radical trachelectomy in selected cases where fertility is desired^42^, contributing to the falling probability of hysterectomy overall and radical hysterectomy over the study period.

The earlier peak for total and subtotal hysterectomy at 45–49 years found in our study, is consistent with that of estimates in the United States^43^, Denmark^23^ and Brazil^36^. In a cross-sectional analysis of women who had undergone a hysterectomy in the city of Mainz and the Mainz-Bingen region in Germany, the probability of having a hysterectomy was highest in the 45–49-year age group^44^. The aetiology of benign gynaecological conditions such as fibroids, endometriosis, adenomyosis and endometrial hyperplasia that affect women in the reproductive age group is linked to dysregulation in reproductive hormones; estrogen and progesterone^45^. Ultrasound studies showed an increasing prevalence of uterine fibroids regardless of symptoms with increasing premenopausal age^46^. In a cohort study conducted in California, the 45–49-year age group had the highest incidence rate of uterine fibroids^47^. Even for those with earlier symptoms, the desire to preserve fertility might lead to the initial use of conservative options to fulfill patients' wishes, thereby pushing the need for hysterectomy to later ages. In a retrospective study of surgical treatments for fibroids, patients over 40 years preferred hysterectomy^48^, possibly because most women have no further intentions of preserving their fertility by then.

In Germany, the median age for endometrial cancer diagnosis is 68^41^. Since radical hysterectomy is generally indicated in invasive cervical^42^ and advanced endometrial cancers^49^ and endometrial cancer is the most common gynaecological cancer in Germany, it is not surprising that the highest rate for radical hysterectomy was in the 65–69 years age group. The higher incidence of gynaecological malignancies in older ages may also contribute to higher total hysterectomy rates after 60 years. With about 70% of endometrial cancers being diagnosed at an early stage^41^, these are, however, more likely to be treated surgically with total hysterectomy and bilateral adnexectomy, as strongly recommended by the German S3 endometrial cancer guidelines for endometrial cancer stage^49^.

Although this paper presents an age-period-cohort analysis of the national hysterectomy incidence trends in Germany, the relatively short study period may make it difficult to observe cohort effects and introduce data artifacts. Since this is an ecological study, analysis and interpretations are based on a population level and might not hold for all regions and their variations in Germany. In 2014, Stang and colleagues reported higher hysterectomy rates in West Germany compared to the East from six population cohorts from 1997 to 2006^50^. A small proportion of hysterectomies are performed as outpatient cases, which are not captured in the Diagnoses Related Group data^51^.

The dynamics of the trends of hysterectomy subtypes in Germany are changing and has important implications for clinical practice. With increasing subtotal hysterectomy rates, the number of women who have undergone hysterectomy but have a cervix and, therefore, are still at risk of cervical cancer also increases. There is a need to educate this population on the need to continue cervical cancer screening. Continuous monitoring of trends is needed to detect these changing dynamics and guide practice accordingly.

### Supplementary Information

Supplementary Table 1.

Supplementary Table 2.

## Data Availability

The data underlying this article are publicly available from the Genesis Online Database of the German Federal Statistical Office at Federal Statistical Office Germany—GENESIS-Online: Database of the<br/>Federal Statistical Office of Germany (destatis.de). Using the OPS codes for hysterectomy, the hysterectomy dataset were retrieved from this link: Federal Statistical Office Germany—GENESIS-Online: Table retrieval (destatis.de). The data on the population statistics were retrieved from this link: Statistisches Bundesamt Deutschland—GENESIS-Online: Tabelle abrufen (destatis.de).

## References

[1] Lefebvre G, Allaire C, Jeffrey J (2002). SOGC clinical guidelines. Hysterectomy. J Obstet Gynaecol Can..

[2] Prütz F, Knopf H, von der Lippe E, Scheidt-Nave C, Starker A, Fuchs J (2013). Prävalenz von Hysterektomien bei Frauen im Alter von 18 bis 79 Jahren.

[3] Gesundheitsberichtserstattung des Bundes. *Operations and Procedures of Full-Time Patients in Hospitals (Place of Residence/Treatment). Classification: Years, Region, Age, Sex*. (2020). https://www.gbe-bund.de/gbe/pkg_isgbe5.prc_menu_olap?p_uid=gast&p_aid=22282561&p_sprache=E&p_help=2&p_indnr=662&p_version=1&p_ansnr=88389803.

[4] AQUA: Institut für angewandte Qualitätsförderung und Forschung im Gesundheitswesen GmbH. *Bundesauswertung zum Erfassungsjahr 2012. 15/1 Gynäkologische Operationen*. (AQUA GmbH, 2013).

[5] Stang A, Merrill RM, Kuss O (2011). Hysterectomy in Germany: A DRG-based nationwide analysis, 2005–2006. Dtsch. Arztebl. Int..

[6] Boosz AS, Reimer P, Matzko M, Römer T, Müller A (2014). The conservative and interventional treatment of fibroids. Dtsch. Arztebl. Int..

[7] Stratopoulou CA, Donnez J, Dolmans MM (2021). Conservative management of uterine adenomyosis: Medical vs surgical approach. J. Clin. Med..

[8] Deutsche Gesellschaft für Gynäkologie und Geburtshilfe. *Indikation und Methodik der Hysterektomie bei benignen Erkrankungen* (AWMF Registernummer 015/077, Leitlinienklasse 3). (2015).

[9] Neis KJ, Zubke W, Fehr M, Römer T, Tamussino K, Nothacker M (2016). Hysterectomy for benign uterine disease. Dtsch. Arztebl. Int..

[10] Kim ML, Seong SJ (2013). Clinical applications of levonorgestrel-releasing intrauterine system to gynecologic diseases. Obstet. Gynecol. Sci..

[11] Prütz F, von der Lippe E (2014). Hysterektomie.

[12] Carstensen B (2007). Age–period–cohort models for the Lexis diagram. Stat. Med..

[13] Blanchard RD, Wachs JB, Wachs M (1977). Distinguishing aging, period and cohort effects in longitudinal studies of elderly populations. Socio-Econ. Plann. Sci..

[14] Lisonkova S, Lavery JA, Ananth CV (2016). Temporal trends in obstetric trauma and inpatient surgery for pelvic organ prolapse: An age-period-cohort analysis. Am. J. Obste. Gynecol..

[15] Salvatori A, Andreano A, Decarli A, Russo AG (2022). Age–period–cohort effects in utilization of diagnostic procedures leading to incidental colorectal cancer detection. Eur. J. Cancer Prev..

[16] Bundesamt, S. *Fallpauschalenbezogene Krankenhausstatistik (DRG-Statistik) Operationen und Prozeduren der Vollstationären Patientinnen und Patienten in Krankenhäusern (4-Steller)*. (2022). https://www.destatis.de/DE/Themen/Gesellschaft-Umwelt/Gesundheit/Krankenhaeuser/_inhalt.html#_c248rm3qh. Accessed 30th Nov 2022.

[17] 12411-0003: Population: Germany, Reference Date, Sex [Database on the Internet] (2022). https://www-genesis.destatis.de/genesis/online#astructure.

[18] Clayton D, Schifflers E (1987). Models for temporal variation in cancer rates. I: Age-period and age-cohort models. Stat. Med..

[19] Edler KM, Tamussino K, Fülöp G (2017). Rates and routes of hysterectomy for benign indications in Austria 2002–2014. Geburtshilfe Frauenheilkd..

[20] Gante I, Medeiros-Borges C, Águas F (2017). Hysterectomies in Portugal (2000–2014): What has changed?. Eur. J. Obstet. Gynecol. Reprod. Biol..

[21] Lepka P, Jędryka M, Misiek M, Matkowski R (2018). Hysterectomy in Poland between 2011 and 2016: Changing trends in the surgical approach to hysterectomy. Ginekol. Pol..

[22] Stewart EA, Shuster LT, Rocca WA (2012). Reassessing hysterectomy. Minn. Med..

[23] Lycke KD, Kahlert J, Damgaard R, Mogensen O, Hammer A (2021). Trends in hysterectomy incidence rates during 2000–2015 in Denmark: Shifting from abdominal to minimally invasive surgical procedures. Clin. Epidemiol..

[24] Ebner F, de Gregorio N, Lato C (2020). Choosing a surgical access point for hysterectomy: A paradigm shift over a 10-year span. Front. Med..

[25] Müller A, Thiel F, Jud SM (2007). Hysterektomie: Was ist zeitgemäß?. Geburtshilfe Frauenheilkd..

[26] Wong WS, Lim CED (2013). Factors influencing the choice of hysterectomy approach for the management of fibroid uterus. Gynecol. Minim. Invas. Ther..

[27] Jenkins TR (2004). Laparoscopic supracervical hysterectomy. Am. J. Obstet. Gynecol..

[28] van Evert JS, Smeenk JMJ, Dijkhuizen FPHLJ, de Kruif JH, Kluivers KB (2010). Laparoscopic subtotal hysterectomy versus laparoscopic total hysterectomy: A decade of experience. Gynecol. Surg..

[29] Kilkku P, Grönroos M, Hirvonen T, Rauramo L (1983). Supravaginal uterine amputation vs hysterectomy: Effects on libido and orgasm. Acta Obstet. Gynecol. Scand..

[30] Saini J, Kuczynski E, Gretz HF, Sills ES (2002). Supracervical hysterectomy versus total abdominal hysterectomy: Perceived effects on sexual function. BMC Womens Health..

[31] Aleixo GF, Fonseca MCM, Bortolini MAT, Brito LGO, Castro RA (2019). Total versus subtotal hysterectomy: Systematic review and meta-analysis of intraoperative outcomes and postoperative short-term events. Clin. Ther..

[32] Lethaby A, Mukhopadhyay A, Naik R (2012). Total versus subtotal hysterectomy for benign gynaecological conditions. Cochrane Database Syst. Rev..

[33] Renner SP, Beckmann MW (2016). S3-leitlinie hysterektomie, indikation und methodik. Bayerisches Ärzteblatt..

[34] Desai VB, Wright JD, Lin H (2019). Laparoscopic hysterectomy route, resource use, and outcomes: Change after power morcellation warning. Obstet. Gynecol..

[35] Hakkarainen J, Nevala A, Tomás E (2021). Decreasing trend and changing indications of hysterectomy in Finland. Acta Obstet. Gynecol. Scand..

[36] Augusto CF, Caraça DB, Podgaec S (2021). Epidemiological analysis of hysterectomies performed at the public health system in the largest Brazilian city. Rev. Assoc. Méd. Bras..

[37] Sutton C (1997). Hysterectomy: A historical perspective. Baillieres Clin. Obstet. Gynaecol..

[38] Fox J, Remington P, Layde P, Klein G (1999). The effect of hysterectomy on the risk of an abnormal screening Papanicolaou test result. Am. J. Obstet. Gynecol..

[39] Storm HH, Clemmensen IH, Manders T, Brinton LA (1992). Supravaginal uterine amputation in Denmark 1978–1988 and risk of cancer. Gynecol. Oncol..

[40] Bujan Rivera J, Klug SJ (2018). Cervical cancer screening in Germany. Bundesgesundheitsblatt Gesundheitsforschung Gesundheitsschutz..

[41] Robert Koch-Institut. *Gesellschaft der Epidemiologischen Krebsregister in Deutschland e.V. Krebs in Deutschland 2015/2016*. (2019). 10.25646/5977

[42] Leitlinienprogramm Onkologie. *(Deutsche Krebsgesellschaft, Deutsche Krebshilfe, AWMF): S3-Leitlinie Diagnostik, Therapie und Nachsorge der Patientin mit Zervixkarzinom, Kurzversion, 2.2 2022, AWMF-Registernummer: 032/033OL* (2022).

[43] Wright JD, Herzog TJ, Tsui J (2013). Nationwide trends in the performance of inpatient hysterectomy in the United States. Obstet. Gynecol..

[44] Tanaka LF, Schoffer O, König J, Weyer-Elberich V, Blettner M, Klug SJ (2023). Changes in the probability of hysterectomy in the city of Mainz and Mainz-Bingen region, Germany. BMC Public Health..

[45] MacLean JA, Hayashi K (2022). Progesterone actions and resistance in gynecological disorders. Cells..

[46] Wise LA, Laughlin-Tommaso SK (2016). Epidemiology of uterine fibroids: From menarche to menopause. Clin. Obstet. Gynecol..

[47] Templeman C, Marshall SF, Clarke CA (2009). Risk factors for surgically removed fibroids in a large cohort of teachers. Fertil. Steril..

[48] Hackethal A, Brüggmann D, Leis A, Langde S, Stillger R, Münstedt K (2009). Surgical management of uterine fibroids in Hesse, Germany, between 1998 and 2004. Fertil. Steril..

[49] Leitlinienprogramm. *(Deutsche Krebsgesellschaft DK, AWMF Online: S3-Leitlinie Endometriumkarzinom, Langversionversion 2.0, September 2022, AWMF-Register-Nummer: 032/034-OL*.

[50] Stang A, Kluttig A, Moebus S (2014). Educational level, prevalence of hysterectomy, and age at amenorrhoea: A cross-sectional analysis of 9536 women from six population-based cohort studies in Germany. BMC Womens Health..

[51] Salfelder A, Lueken RP, Gallinat A (2007). Hysterektomie als standardeingriff in der tagesklinik: Ein wagnis? Erfahrungen mit ambulanten hysterektomien. Frauenarzt..

