# Supplementary material for: An age-period-cohort analysis of hysterectomy incidence trends in Germany from 2005 to 2019
Source: Sci Rep. 2024 Jul 2;14:15110. doi: 10.1038/s41598-024-66019-8 (PMC11220048; doi:10.1038/s41598-024-66019-8)
Supplement: Supplementary file 1 — Supplementary Table 1. [file 41598_2024_66019_MOESM1_ESM.docx]

**An Age-Period-Cohort Analysis of Hysterectomy Incidence Trends in Germany from 2005 to 2019**

Authors: Gifty Baffour Awuah, MBChB MSc^1^, Gunther Schauberger, PhD^1^, Prof. Stefanie J. Klug, PhD MPH^1^, Luana Fiengo Tanaka, PhD^1^

Supplementary Table 1. Wald Chi-Square tests for estimable functions in the Age-Period-Cohort model. Hysterectomy incidence by subtype in Germany 2005-2019.

| **Null hypothesis** | **Implications** | **Degrees of freedom** | **Total hysterectomy** | | **Subtotal Hysterectomy** | | **Radical Hysterectomy** | |
| --- | --- | --- | --- | --- | --- | --- | --- | --- |
|  |  |  | **X^2^** | P value | **X^2^** | P value | **X^2^** | P value |
| Net drift = 0 | Fitted temporal trends are stable (i.e., flat with no change) over time.  Fitted longitudinal and cross-sectional age curves are proportional. | 1 | 1044.69 | <0.01 | 775.61 | <0.01 | 1597.08 | 0.00 |
| All age deviations = 0 | Fitted longitudinal and cross-sectional age curves are log-linear (i.e., log-additive). | 12 | 3089.13 | 0.00 | 4517.64 | 0 | 1775.23 | 0.00 |
| All period deviations = 0 | Fitted temporal trends and period rate ratios are log-linear (i.e., log-additive). | 1 | 69.89 | <0.01 | 821.65 | <0.01 | 240.31 | <0.01 |
| All cohort deviations = 0 | Cohort rate ratios are log-linear; all local drifts equal the net drift. | 14 | 157.37 | <0.01 | 151.38 | <0.01 | 59.14 | <0.01 |
| All period rate ratios = 1 | Net drift is 0 and fitted temporal trends are constant;  Cross-sectional age curve describes age incidence pattern in every period. | 2 | 1084.98 | <0.01 | 1398.80 | <0.01 | 1760.46 | 0.00 |
| All cohort rate ratios = 1 | Net drift is 0 and all local drifts are 0;  Longitudinal age curve describes age incidence pattern in every cohort. | 15 | 2709.36 | 0.00 | 2380.43 | 0 | 2521.79 | 0.00 |
| All local drifts = the net drift | Temporal patterns are the same in every age group. | 14 | 157.37 | <0.01 | 151.38 | <0.01 | 59.14 | <0.01 |
