# Supplementary material for: An age-period-cohort analysis of hysterectomy incidence trends in Germany from 2005 to 2019
Source: Sci Rep. 2024 Jul 2;14:15110. doi: 10.1038/s41598-024-66019-8 (PMC11220048; doi:10.1038/s41598-024-66019-8)
Supplement: Supplementary file 2 — Supplementary Table 2. [file 41598_2024_66019_MOESM2_ESM.docx]

**An Age-Period-Cohort Analysis of Hysterectomy Incidence Trends in Germany from 2005 to 2019**

Authors: Gifty Baffour Awuah, MBChB MSc^1^, Gunther Schauberger, PhD^1^, Prof. Stefanie J. Klug, PhD MPH^1^, Luana Fiengo Tanaka, PhD^1^

Supplementary Table 2: Age, Period and Cohort estimates from the APC analysis for all hysterectomies and subtypes. Germany 2005-2019.

| **Variable**  **Age** | **Total hysterectomy** | | **Subtotal hysterectomy** | | **Radical hysterectomy** | |
| --- | --- | --- | --- | --- | --- | --- |
|  | **Rate*** | **95% CI** | **Rate*** | **95% CI** | **Rate*** | **95% CI** |
| 20-24 | 2.71 | 1.64 – 4.47 | 1.06 | 0.63 - 1.77 | 0.47 | 0.36 - 0.62 |
| 25-29 | 22.24 | 18.51 – 26.73 | 4.51 | 3.59 - 5.65 | 2.39 | 2.11 - 2.71 |
| 30-34 | 97.18 | 86.28 – 109.45 | 19.74 | 17.29 - 22.53 | 7.74 | 7.09 - 8.45 |
| 35-39 | 280.59 | 254.42 – 309.46 | 64.72 | 58.42 - 71.70 | 14.26 | 13.20 - 15.40 |
| 40-44 | 545.95 | 501.88 – 593.89 | 140.39 | 128.31 - 153.62 | 18.97 | 17.67 – 20.36 |
| 45-49 | 608.63 | 565.70 – 654.82 | 151.30 | 138.38 - 165.44 | 23.00 | 21.55 - 24.54 |
| 50-54 | 384.04 | 359.48 – 410.28 | 70.94 | 64.19 - 78.38 | 28.77 | 27.12 – 30.52 |
| 55-59 | 232.77 | 218.43 – 248.06 | 21.35 | 18.91 - 24.10 | 35.83 | 33.96 – 37.81 |
| 60-64 | 237.00 | 223.06 – 251.81 | 15.26 | 13.26 - 17.56 | 39.64 | 37.74 - 41.64 |
| 65-69 | 265.62 | 250.76 – 281.36 | 14.12 | 12.11 - 16.47 | 40.63 | 38.84 - 42.52 |
| 70-74 | 290.80 | 274.30 – 308.29 | 13.72 | 11.65 - 16.15 | 39.98 | 38.27 - 41.77 |
| 75-79 | 278.26 | 260.74 – 296.97 | 11.64 | 9.77 - 13.85 | 32.96 | 31.41 - 34.60 |
| 80-84 | 218.59 | 200.24 - 238.63 | 6.67 | 5.32 - 8.36 | 20.17 | 18.87 - 21.57 |
| 85+ | 144.47 | 12713 – 165.25 | 2.10 | 1.43 - 3.07 | 7.22 | 6.43 – 8.10 |
| **Period** | **RR** | **95% CI** | **RR** | **95% CI** | **RR** | **95% CI** |
| 2005-2009 | 1.39 | 1.21 - 1.28 | 0.43 | 0.41 - 0.45 | 1.15 | 1.12 - 1.17 |
| 2010-2014 | 1.00 | 1.00 - 1.00 | 1.00 | 1.00 - 1.00 | 1.00 | 1.00 - 1.00 |
| 2015-2019 | 0.66 | 0.64 - 0.68 | 1.09 | 1.05 - 1.14 | 0.62 | 0.60 - 0.63 |
| **Cohort** | **RR** | **95% CI** | **RR** | **95% CI** | **RR** | **95% CI** |
| Up to 1920 | 6.05 | 4.78 – 7.66 | 0.03 | 0.01 -0.06 | 16.56 | 13.54 - 20.26 |
| 1921-1925 | 5.56 | 4.72 – 6.55 | 0.03 | 0.02 -0.04 | 10.25 | 9.00 - 11.67 |
| 1926-1930 | 4.94 | 4.33 – 5.64 | 0.03 | 0.02 -0.04 | 7.67 | 6.92 - 8.48 |
| 1931-1935 | 3.78 | 3.38 – 4.23 | 0.04 | 0.04 -0.06 | 5.57 | 5.11 - 6.08 |
| 1936-1940 | 3.08 | 2.78 – 3.40 | 0.08 | 0.06 - 0.10 | 4.14 | 3.83 - 4.48 |
| 1941-1945 | 2.33 | 2.14 – 2.54 | 0.16 | 0.14 - 0.20 | 2.79 | 2.61 - 3.00 |
| 1946-1950 | 1.75 | 1.63 – 1.88 | 0.31 | 0.28 – 0.35 | 1.86 | 1.76 - 1.98 |
| 1951-1955 | 1.38 | 1.31 - 1.45 | 0.58 | 0.54 – 0.62 | 1.40 | 1.33 - 1.47 |
| 1956-1960 | 1.00 | 1.00 - 1.00 | 1.00 | 1.00 - 1.00 | 1.00 | 1.00 - 1.00 |
| 1961-1965 | 0.69 | 0.66 – 0.72 | 1.58 | 1.52 - 1.65 | 0.75 | 0.71 - 0.79 |
| 1966-1970 | 0.46 | 0.44 – 0.49 | 2.30 | 2.19 - 2.41 | 0.56 | 0.52 - 0.60 |
| 1971-1975 | 0.28 | 0.27 – 0.30 | 3.02 | 2.83 – 3.21 | 0.39 | 0.36 - 0.43 |
| 1976-1980 | 0.18 | 0.17 - 0.20 | 4.13 | 3.75 – 4.54 | 0.31 | 0.28 - 0.34 |
| 1981-1985 | 0.13 | 0.12 - 0.16 | 6.09 | 5.13 – 7.24 | 0.28 | 0.24 - 0.33 |
| 1986-1990 | 0.10 | 0.07 - 0.15 | 8.90 | 6.22 – 12.74 | 0.21 | 0.17 - 0.28 |
| 1991-1995 | 0.09 | 0.03 - 0.25 | 5.96 | 2.10 – 16.91 | 0.18 | 0.10 - 0.31 |

*Rate per 100,000 women, CI: Confidence Interval, RR: Rate ratio
